# The role of parents in the care for adolescents suffering from emotional and behavioral problems

**DOI:** 10.3389/fpsyg.2022.1049247

**Published:** 2022-12-22

**Authors:** Jaroslava Mackova, Zuzana Dankulincova Veselska, Andrea Madarasova Geckova, Danielle E. M. C. Jansen, Jitse P. van Dijk, Sijmen A. Reijneveld

**Affiliations:** ^1^Department of Health Psychology and Research Methodology, Faculty of Medicine, Pavol Jozef Safarik University, Kosice, Slovakia; ^2^Olomouc University Social Health Institute, Palacky University Olomouc, Olomouc, Czechia; ^3^Department of Community and Occupational Medicine, University Medical Center Groningen, University of Groningen, Groningen, Netherlands

**Keywords:** adolescents, psychosocial care, perceptions of care providers, role of parents, qualitative analyses

## Abstract

**Background:**

Parents play an important role in the psychosocial care of their children. Previous research has primarily examined their role in care entry, whereas evidence on their role in other stages of the care process is scarce and lacking particularly in regard to the perspective of care providers. Our aim was therefore to examine how psychosocial care providers perceive the role of parents in the process of psychosocial care for adolescents.

**Methods:**

We used data from 25 semi-structured interviews with psychosocial care providers on the roles of parents in the care process. We analyzed data using consensual qualitative research and thematic analysis.

**Results:**

Four main themes were identified: (1) parents as a source of adolescents’ problems; (2) parents trying to escape from responsibility for adolescents with problems; (3) parents as an active part of the care for adolescents; and (4) parents as a barrier to effective care.

**Conclusion:**

Psychosocial care providers should specifically address the role of the parents in psychosocial care to improve outcomes. Specific interventions are needed to support the involvement of parents in care, as their role is important. Moreover, professionals can be better trained in working with multi-problem families also to resolve some of the negative perceptions of these parents.

## Introduction

A significant number (ranging from 13 to 17%) of adolescents worldwide suffer from emotional and behavioral problems (EBP; Barkmann and Schulte-Markwort, 2012; Philipp et al., 2018; Geckova et al., 2019; Yang et al., 2019). Emotional and behavioral problems are defined as “behaviors or emotions that deviate so much from the norm that they interfere with the child’s own growth and development and/or the lives of others” (Cooper, 2011 p. 71–72). Emotional problems include depression, withdrawal, social phobia, specific phobias, anxiety, post-traumatic stress disorder, obsessive–compulsive disorder, poor self-esteem, as well as feelings of inferiority, self-consciousness, shyness, hypersensitivity, and somatic complaints or eating disorders. Behavioral problems, on the other hand, include defiance, impulsivity, disruptiveness, aggression, antisocial behavior, and over activity as well as problems with attention and self-regulation, such as temper tantrums, substance abuse, and bullying (Achenbach et al., 1991; Bornstein et al., 2013; Ogundele, 2018). However, not all adolescents – not even those from high-income countries – receive adequate help and treatment (Burns et al., 1995; Flisher et al., 1997; Zwaanswijk et al., 2003; Paclikova et al., 2020). Various factors may influence whether adolescents receive help or not, such as ethnicity and insurance (Kataoka et al., 2002; Ngui and Flores, 2007), low socioeconomic status (Flisher et al., 1997), or family factors, such as parental psychopathology, substance abuse, and increased number of siblings (Cornelius et al., 2001). Moreover, parents play an essential role in many instances (e.g., in access, adherence, and outcomes) of the care provided to their children, as they are legally responsible for their children and legally should be involved at least to a certain age of the adolescent.

As adolescents cannot make decisions about their health on their own parents play a crucial role in the whole process of providing psychosocial care to their children. The provision of care starts with the ability of parents to recognize problems and access the care system, through the willingness to cooperate and adhere to the treatment until the outcomes of the care. Evidence on parental roles in psychosocial care for adolescents focused mostly on help-seeking behavior and access to care (e.g., Glascoe and Dworkin, 1995; Logan and King, 2001; Wahlin and Deane, 2012), and much less on other stages of the care process. Glascoe and Dworkin (1995) recognized parents as an important source of information for professionals in the early detection of problems in their children. Their inability to recognize the seriousness of adolescent problems as well as to convince the adolescent to seek help have been considered to be key barriers in finding professional help (Oh and Bayer, 2015). Although adolescents facing problems seek help from different sources such as peers, friends, or teachers, Wahlin and Deane (2012) found that parents are the most influential factor in the help-seeking behavior in the case of adolescents. Radovic et al. (2015) confirmed that parents play a crucial role in accessing mental health care services. Adding to that, Logan and King (2001) proposed the model of a parent-mediated pathway to mental health services for adolescents, in which parents have a crucial role in help-seeking and go through the five following stages: (1) Parents gain the initial awareness of the adolescent’s distress, (2) Parents recognize that the problem is severe and requires attention, (3) Parents consider options for helping their children, (4) Parents develop an intention to seek mental health services; and (5) Parents attempt to seek appropriate mental health services. After these steps, the adolescent finally obtains care.

A few studies focused on other stages of care rather than access, the rare studies, e.g., showing the important role of parents in regular attendance and adherence to child psychotherapy (Nock and Ferriter, 2005) or pharmacotherapy (O’Brien et al., 2013). Another study showed that parents perceived the importance of their own role in the recovery of adolescents, as they considered their relationship with the adolescent as the most important external factor in the recovery (Kelly and Coughlan, 2019). Adding to that, the meta-analytical review of Dowell and Ogles (2010) and a meta-analysis of Sun et al. (2019) showed that including parents in the treatment of their children brought an additional benefit to this treatment. To fill the gaps in knowledge as indicated, our study could provide evidence regarding the roles of parents in the whole process of care, from the identification of problems in adolescents through entering care to attending care and adhering to it, i.e., wider than only regarding the help-seeking behavior in stricter sense.

A major issue regarding parental roles concerns the perspective of professionals on the role of parents, as professional–parent cooperation is very important in the psychosocial care for adolescents. One of the few studies aiming to explore this issue (Radovic et al., 2015) focused on primary care providers’ perception of the role of parents of adolescents suffering from depression. They found that the role of parents is crucial in accessing mental health care services but also that parents may create obstacles to care by their unwillingness to accept a diagnosis, family dysfunction, or trauma in the past in the adolescent. Further evidence on the role of parents from the perspective of care providers is mostly lacking but is greatly needed. Therefore, we aimed to assess the perception of psychosocial care providers regarding the role of parents in the process of care.

## Materials and methods

### Design

We performed a qualitative study that was embedded in the Slovak Care4Youth (C4Y) study mapping the system of psychosocial care provided for adolescents with EBP and its characteristics from the perspective of care providers. We performed this qualitative study in three phases. First, we established a protocol and outlined the topic guide for semi-structured interviews. Second, we collected the data using semi-structured interviews from May 2017 until November 2018. Last, we analyzed the acquired data using a combination of the consensual qualitative research (CQR) methodology (Hill et al., 1997) and thematic analysis (Braun and Clarke, 2006). The study was approved by the Ethics Committee of the Medical Faculty at Pavol Jozef Safarik University in Kosice (protocol 2 N/2015).

### Study setting, sampling, and participants

Our sample consisted of professionals working with adolescents with EBP. The system of psychosocial care in Slovakia comprises three main types of care – preventive-counselling, social and mental health care. Preventive-counselling care is organized predominantly by the Ministry of Education, Science, Research and Sport and provides predominantly diagnostics and counselling for adolescents facing problems associated with the school environment. The second type, social care, is predominantly organized by the Ministry of Labor, Social Affairs and Family, though it also includes non-profit organizations. This type of care includes first the Office of Social and Legal Protection of Children and the Office of the Social Curator, which should prevent crisis in the family, protect the rights and interests of children and prevent deepening and repeating of disorders of healthy development. Second, it includes professionals from the national project of deinstitutionalization of care, which provide social work, counselling, and field work with the family with the aim of preventing the removal of a child from the family. Last, it includes non-profit organizations providing a broad range of services, usually including social counselling and social work with whole families in a difficult situations (such as low-income families, families in divorce, children with different problems such as EBP, etc.). The third type of care, mental health care, is predominantly organized by the Ministry of Health and includes outpatient care involving psychiatrists providing pharmacotherapy and treatment for adolescents, as well as clinical psychologists providing diagnostics and psychotherapy. Moreover, mental health care includes inpatient care provided by psychiatric hospitals for acute care and psychiatric long-stay hospitals for long-term care.

We obtained our sample of care providers following a two-step sampling to reach a representative sample of all types of institutions providing preventive-counselling, social or mental health care in Slovakia. First, we identified and chose representatives of all types of institutions in preventive-counselling, social and mental health care in the Košice region, Slovakia. The project researchers contacted the selected institutions, and all 17 approached institutions agreed to participate in the study. Second, within each institution, all care providers working with adolescents were asked to participate in the study, and all 49 care providers agreed. They received information about the study, were informed about the voluntary and anonymous basis of the participation, and provided informed consent.

### Procedure and measures

We performed semi-structured interviews to gain insight into different aspects of the care for adolescents with EBP from the perspective of professionals. The interviews lasted approximately 60–120 min and took place in the institutions where the care providers work; they were conducted in the Slovak language and were recorded. Each interview was conducted by one to three interviewers (the principal investigator conducted all the interviews, others alternated). All interviewers were trained researchers. The research team consisted of researchers with a background in psychology and social work on different levels of their academic careers with various experiences from their previous work in the system of care.

In line with the purpose of this study, we asked questions about the roles of the parent in this process (e.g., how the client/family entered care) and included them in the analysis. These questions were embedded in a wider set of questions about different aspects of the care provided by the institution, including questions on the competencies of providers, setup of the work with clients, the methods they used in their work and their theoretical background, cooperation with other institutions, perceived barriers and facilitators of care, and provider’s suggestions about what might help to improve the care for adolescents.

We conducted 25 individual and group interviews with care providers in the period between May 2017 and November 2018, involving one to four providers per interview. We performed individual or group interviews depending on the type of institution. Group interviews were performed in institutions with multidisciplinary teams. This approach did not affect the data collected and analyzed as the researcher conducting the interviews ensured that all respondents expressed their opinions also in group interviews similar to in individual interviews. As some of those care providers were at the same time head or chief of the institution, in the case of institutions with a hierarchical organizational structure, the head or the chief of the institution was interviewed separately from the employees to create a space for open expression of attitudes otherwise potentially hindered by the power imbalance. The sample consisted of 49 care providers from the 17 institutions that provided preventive-counselling, social and health care for adolescents with EBP, with the following backgrounds: 20 psychologists, 18 social workers, 4 child psychiatrists, 4 teachers, 1 educational counsellor, 1 pedagogue for adolescents with special needs and 1 nurse. Some of the care providers were at the same time head or chief of their institution. Most of the respondents were female and they had spent from 1 to 24 years in their current position (for a detailed overview, see Table 1).

**Table 1:** Characteristics of the sample by type of care, participant’s profession, gender and years of practice in the current position.

Interview	Type of care	Participants profession	Gender	Years of practice in current position
1	Preventive-counselling care	R1: Teacher	R1: Female	R1: 17
		R2: Teacher	R2: Female	R2: 19
2	Preventive-counselling care	R1: Educational counsellor	R1: Female	R1: 8
3	Preventive-counselling care	R1: Chief of the institution/Psychologist	R1: Female	R1: 11
		R2: Psychologist	R2: Female	R2: 3
4	Preventive-counselling care	R: Chief of the institution /Pedagogue for special needs R2: Psychologist	R1: Female	R1: 12
			R2 Female	R2: 3
5	Preventive-counselling care	R1: Chief of the institution	R1: Female	R1: 10
		R2: Psychologist	R2: Female	R2: 8
		R3: Psychologist	R3: Female	R3: 5
6	Preventive-counselling care	R1: Psychologist	R1: Female	R1: 2
7	Preventive-counselling care	R1: Teacher/ Pedagogue for special needs	R1: Female	R1: 11
		R2: Social worker	R2: Female	R2: 1
		R3: Social worker	R3: Female	R3: 5
8	Preventive-counselling care	R1: Chief of the institution /Teacher	R1: Male	R1: 19
9	Social care	R1: Chief of the institution/Psychologist	R1: Female	R1: 15
		R2: Psychologist	R2: Female	R2: 1
10	Social care	R1: Chief of the institution/Social worker	R1: Female	R1: 15
		R2: Social worker	R2: Male	R2: 5
11	Social care	R1: Psychologist	R1: Female	R1: -
		R2: Psychologist	R2: Female	R2: -
		R3: Social worker	R3: Female	R3: -
12	Social care	R1: Social worker	R1: Female	R1: 1
		R2: Social worker	R2: Male	R2: 1
13	Social care	R1: Psychologist	R1: Female	R1: 2
		R2: Social worker	R2: Female	R2: 2
14	Social care	R1: Psychologist	R1: Female	R1: 1
		R2: Social worker	R2: Female	R2: 1
15	Social care	R1: Psychologist	R1: Female	R1: 24
		R2: Psychologist	R2: Female	R2: 12
16	Social care	R1: Social worker	R1: Female	R1: 7
		R2: Social worker	R2: Female	R2: 5
17	Social care	R1: Chief of the institution / Social worker	R1: Female	R1: 23
		R2: Chief of the institution / Social worker	R2: Male	R2: 19
18	Social care	R1: Psychologist	R1: Female	R1: 8
		R2: Social worker	R2: Female	R2: 5
19	Health care	R1: Psychologist	R1: Female	R1: 10
		R2: Psychologist	R2: Female	R2: 10
20	Health care	R1: Chief of the institution/Social worker	R1: Female	R1: 20
21	Health care	R1: Chief of the institution/Psychiatrist	R1: Female	R1: 3
22	Health care	R1: Psychologist	R1: Female	R1: 2
		R2: Psychiatrist	R2: Female	R2: 21
		R3: Nurse	R3: Female	R3: 14
		R4: Social worker	R4: Female	R4: 12
23	Health care	R1: Psychologist	R1: Female	R1: 4
24	Health care	R1: Psychiatrist	R1: Female	R1: 8
25	Health care	R1: Chief of the institution/Psychiatrist	R1: Female	R1: 3
		R2: Psychologist	R2: Female	R2: 1

### Data handling, analysis, and reporting

We handled the data by first transcribing the interviews verbatim in the Slovak language. The transcriptions were checked by the principal investigator to ensure the accuracy of the transcription process. Next, we coded the data using the CQR methodology to provide a basis for thematic analysis. Three teams of coders (one team per type of care providers) were involved. Each team consisted of four to five coders and one or two auditors (professors of psychology). Two main coders (the principal investigator and a PhD student) read and coded all the interviews from all three types of the system of care. Other members of the teams were chosen based on their experience with the appropriate type of system of care. All coders were researchers who had been trained in CQR. Each team member read the transcripts of the interviews and created codes for parts of the interviews independently. Then, team members met and shared their codes and interpretations with the aim of achieving consensus. In the case of differing opinions, the discussion continued until a consensus was reached. We used the MAXQDA software for the coding and analysis process.

Based on the codes produced in this data handling, we conducted a thematic analysis in accordance to Braun and Clarke (2006). We started by reading and familiarizing ourselves with the data which was followed by data-driven production of initial codes. In the next step we continued with clustering codes regarding the reported roles of parents in any part of the process of care into themes and subthemes. To realize this, we read all the codes and sorted them into groups of subthemes and overarching themes based on the topic they were covering while thinking about the relationship between codes, between themes, and between different levels of themes (e.g., main overarching themes and subthemes within them). The coding and creation of the subthemes and themes were done separately for each of the three types of care. Afterward, we combined the subthemes and themes from preventive-counselling, social and health care and looked for overlaps and differences between them. In cases when we found some differences within the subthemes of the three types of care, we searched for the theme that would cover all the nuances. Finally, an overall thematic map with main overarching themes and subthemes within them representing all types of care was created.

## Results

### Main themes

**Figure 1:**
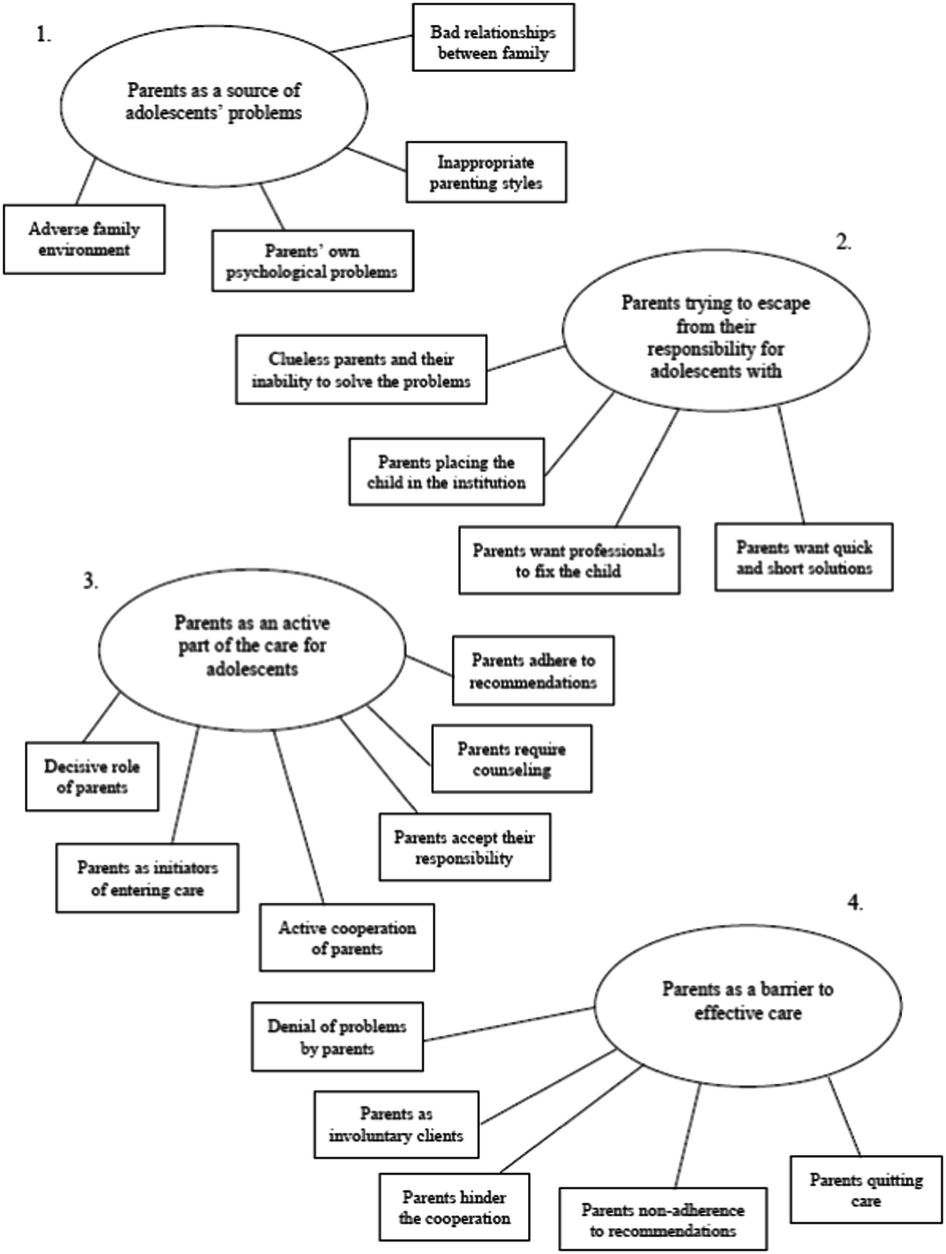
The four main themes with subthemes on perceived roles of parents in care identified from of the perspective of care providers.

We identified four main themes: *1*. *parents as a source of adolescents’ problems; 2*. *parents trying to escape from responsibility for adolescents with problems; 3*. *parents as an active part of the care for adolescents; and 4*. *parents as a barrier to effective care*, which each captured a significant part of the issues raised by respondents. Each theme comprised several separate aspects, which we further label as subthemes. These themes and subthemes as identified are presented in Figure 1, and the findings are presented below, each finding clarified by quotes that represent the core contents of each theme and subtheme.

### Parents as a source of adolescents’ problems

Some providers reported parents as a source of adolescents’ problems due to several reasons that could be distinguished into the following subthemes. An *adverse family environment* was seen as something that could be, from the perspective of care providers, considered a potential source of adolescents’ problems. They reported that usually during the diagnostic process they found that many adolescents with EBP were, to some extent, living in an adverse family environment.

“And so it will show very nicely when you communicate with that child for some time. A really large percentage is in those families where the social background is, so really either divorce, or some problematic domestic relations, or something that will subsequently manifest itself at an older age.”

Adverse family environment as a potential source of adolescents’ problems was by some of the providers attributed to the parents’ own psychological problems. Providers often identified these parental problems as the real causes of the EBP of adolescents.

“Because I think that many times the parents themselves have a problem with themselves. They have existential problems, all kinds of other problems, they don't know how to deal with themselves.”

Parents as a source of adolescents’ problems were mentioned by care providers due to their *inappropriate parenting styles*, such as low supervision, no boundaries being set for the adolescent or being in a submissive position to the adolescent.

“Well, the parent is probably the biggest problem in the whole thing. In the whole problem, because really, even if the parents could have worked differently with the children, the children will not get into such a state. If they paid more attention to them.”

Closely connected to that were also *bad relationships* between family members. In some cases, providers explained that parents were aware of their situation and that this was a reason to seek help.

“They won't go to the child, they won't try, or the parent won't take the first step, like let's put the relationship in order somehow and let's solve things.”

### Parents trying to escape from responsibility for adolescents with problems

Providers reported that in some cases parents tried to escape their responsibility for their own child, an adolescent with problems. Providers described situations that could be distinguished into the several subthemes. The first identified subtheme was *clueless parents and their inability to solve the problems* of their adolescent on their own.

“Because oftentimes, when we then contact the parents, the parent comes and says: "I don't know what to do with him."

“They get so scared, those parents, after something like that happened, and that we somehow have to help them with it and talk to those children, because they, those parents, somehow don't know how to react then.”

This often, from the perspective of care providers, resulted in the subtheme of *parents placing the child in the institution* due to inability of parents to continue caring for the adolescent or even in rejecting the adolescent.

“Some children seem to be already prepared in advance for this situation, that they will go here. That their parents tell them in advance, when they have problems with them, that if they won't change, won't improve, they'll go there.

Moreover, providers mentioned requests of parents for *professionals to fix the child* without any effort and contribution of their own.

“And many times it happens that the parent feels that they hand over the child to us, we fix it and return it repaired and they don't have to do anything anymore.”

Parents were, from the perspective of providers, transferring the responsibilities for adolescents’ problems to the care providers with their wish for *quick and short solutions* for their children’s problems.

“And they are still waiting for some "Potter's wand" that we will flick and the child will change overnight.”

“I rather think that there are those who want to get results quickly. Those like that, they're not even insecure, I don't want to say like that, not even more arrogant, but they've learned that everything is somehow fast, or that they'll get something in return, right away.”

This was interpreted as getting rid of responsibility for the adolescent and his/her treatment at the stage of involvement in care.

### Parents as an active part of the care for adolescents

Care providers reported that active involvement of parents in the whole process of care is crucial; parents play a dominant role in the care process from entering care, *via* assessment of the problems, and in treatment to aftercare. The roles of parents as an active part of the care for adolescents could be split into several subthemes. In the first subtheme, providers identified the significant power of a parent to decide what should be done with his/her child in each of the stages of care mentioned above *via* giving informed consent or not. Therefore, in their perspective, parents play a *decisive role* and may be seen as a facilitator or a barrier (discussed in the next theme) within the whole process of care.

“But when the problems are serious, I have no other option but to contact the parents. They have to agree to it. They have to go to the examination voluntarily.”

“So the parents are always involved to get all the information and get all there is to offer. What they take from it and what they implement is up to them.”

Most of the providers repeatedly recognized parents as being among the main and most often *initiators of entering care*.

“And then there are the families who seek us out themselves, knowing that they can seek help here as well. So they will come on their own.”

Once the parents and their child entered the care, providers often described parents and their *active cooperation*, e.g., setting the goals for care jointly with the professional as being an important part of parental involvement in the ongoing process of providing care.

“When parents are willing to cooperate and the problems are small, improvement will occur. And when they are, and we really get along well with those parents, we know how to correct it and deal with it in time.”

“The parents call to say that there is a problem, they consult on how to proceed further. Most often with psychologists, so what, what would.... They describe a specific situation and want specific advice for a specific situation, usually.”

Next, especially in mental health care, providers identified situations in which parents *accept their responsibility* for the existing situation.

“The parents also receive this information, they receive space to return to how it was when they get divorced because it was actually the result of the unprocessed, actually unprocessed divorce of the parents. So let's talk about it. They realize that "oh, that was a mistake, I did this in a certain way and now I have to do this differently so that I don't actually continue with that bad approach.”

“There is also an effort by the parents to change something, even they seem to admit certain mistakes. “Yeah, maybe I was too directive,” or "I didn't give you enough time," or something, so it's pretty cool in that sense.”

As a result, parents *required counseling* on parenting competencies, setting up a regime at home and improving the ability to build a relationship with the child.

“But there are types of parents who will come and call. There are those who come to talk, to get advice. Because, really, many parents, once they find out that there is a problem, they try to solve it somehow.”

“There are also children from families whose parents are really interested and consult with us and want to help somehow.”

Finally, as the last subtheme, during care and afterwards, providers believed parents play an important role in the *adherence of adolescents to recommendations* made by the professionals.

“Because parents often do not realize how to work with their child. They ask - well, what should we do with that specific, I don't know, disorder that the child has. So we inform them, how to work with them, how to react to them, etc.”

“There are parents who are able to adopt it, they are able to follow some regime rules.”

### Parents as a barrier to effective care

In contrast with actively involved parents described above, professionals also in some cases recognized parents as the potential barrier to care in the whole process of care that could be distinguished into several subthemes.

In the stage of accessing care, parents were perceived as a barrier due to their *denial of problems*, which hindered entering an adolescent into care.

“Because, not every parent wants to see, or will admit that yes, this is my child.... or there is a problem.”

“There are also the stubborn ones. Those who say no way. And they don't realize. Even when I explain to them that the child has a problem. And the problem will drag on with him further, further. Because it will not be removed just like that. By blinking or snapping your fingers. Just not. The parent gets offended, gets up and leaves. It's as if the parents don't want to admit it, they don't realize it.”

Part of care providers also described situations mostly in social care, as a specific subtheme, when entering into care was forced and parents were thus *involuntary client*. In this case, parents were described as being unmotivated to cooperate, having little or no trust in professionals and being afraid that their children would be taken from them.

“In the final cooperation, it will then be reflected that the motivation is not so pure, that "I want to be your client, and I want to work on myself", but rather that "I have to, because they simply put us here".”

“That's basically it, the clients start out as not entirely voluntary clients, I would say, mostly, because actually they are given an obligation to participate.”

Next, care providers reported that some of the parents in process of providing care *hindered the cooperation* with the current provider or with other professionals, as they did not give consent to approach another professional.

“When a parent sees for the first time that we want something from him that he does not agree with, he has to take some action. So very often they are in resistance, but when we don't manage to break it somehow, the parent stops cooperating completely. He does not answer the phone. He avoids, pretends that he is not at home.”

Parents were also perceived by care providers as a barrier due to their *non-adherence to recommendations* for the regimen or care of an adolescent. Some of them were unwilling to change their own behavior when it was detected as part of a problem, had little or no motivation to cooperate and refused the use of medicaments, hospitalization or other suggested procedures.

“Very often, children who are hyperkinetic return because they are set up for treatment, they have some kind of regimen set up. They will leave, let's put it in a compensated state, but in the home environment it is very difficult to maintain the regime.”

“It happens, or has happened, that even a parent violates some treatment regimen. Our specific treatment regimen.”

Due to such ongoing non-cooperation or in the case of dissatisfaction the situation in some cases resulted in *quitting care* initiated by parents rather abruptly and prematurely.

“But sometimes people just leave, even before they solve their problem, they just don't want it anymore. So sometimes it's not just about the fact that it will be resolved, but they just don't want to go on.”

“They come once, twice and stop coming and don't call or pick up the phone anymore, which is what happened to us, that's how it is, the care can't be effective there.”

## Discussion

The aim of this study was to examine and understand the roles parents play in the process of psychosocial care for adolescents from the perspective of providers. We found four main themes regarding parental roles: 1. parents as a source of adolescents’ problems; 2. parents trying to escape from responsibility for adolescents with problems; 3. parents as an active part of the care for adolescents; and 4. parents as a barrier to effective care.

### Parents as a source of adolescents’ problems

We found that from the perspective of care provides, parents may play a role as the original source of the adolescent’s problems; the adolescent might suffer from EBP due to, e.g., substance abuse, violence, divorce, bad relationships, and communication in the family or other issues. This finding is in line with previous research, where an association of adverse childhood experiences (such as violence in the family, substance abuse, divorce, etc.) with the occurrence and number of EBP was found (e.g., Connolly and Kavish, 2019; Lackova Rebicova et al., 2019). Moreover, the study of Nanninga et al. (2015) showed that enrolment into psychosocial care was associated with low family support and poor parenting skills of parents. This issue is also related with the issue of multi-problem families, as children from such families were found to have a high risk of developing psychosocial problems and becoming multi-users of psychosocial care (Pannebakker et al., 2018). This finding points to a serious problem within the care for the adolescents – it is not very efficient to treat an adolescent when problems in the family and its functioning are left unsolved. However, as the meta-analysis of Evenboer et al. (2018) showed, it is not entirely clear which interventions are efficient in treating multi-problem families. From our interviews, we learned that professionals understand that problems have a broader context, and their origin is in the family of adolescent; however, it seemed that they had a lack of competencies for working with clients in such a difficult situation.

### Parents trying to escape from responsibility for adolescents with problems

Next, we found, that providers reported that some parents were unable or unwilling to take care of adolescent with EBP and tried to place the adolescent in the institution. This issue seems to be understudied, but some studies discussed related topics, e.g., O'Sullivan and Russell (2006) described the cycle of blame, in which parents blamed professionals for being not supportive and professionals blamed parents for not being involved in the care. Similarly, Radovic et al. (2015) brought in the perspectives of professionals treating adolescents suffering from depression. They perceived some parents as not being sufficiently involved in care and denying problems, and professionals indirectly blamed them for being a barrier to care. This theme has raised the issue of how professionals deal with their own negative perceptions and attitudes towards parents of adolescents suffering EBP, especially if the parents live in difficult situations and lack parenting and other necessary skills. Moreover, it shows a need to understand why parents are trying to escape from their responsibility. Understanding these reasons and mechanisms may help professionals in working with such parents.

### Parents as an active part of care for adolescents

Care providers recognized the dominant role of a parent within the whole process of care, from entering the care system, through the assessment of the problems of adolescents and the selection of a treatment and the termination of the care. Previous research has shown the importance of the parental role in help-seeking behavior (e.g., Logan and King, 2001; Zwaanswijk et al., 2003; Hoagwood, 2005; Nanninga et al., 2015; Hassett et al., 2018), including the ability to identify problems in children (Glascoe and Dworkin, 1995) and in the recovery of adolescents (Kelly and Coughlan, 2019). Moreover, previous studies have shown that families who had good, collaborative relationship with the care provider were more likely to be willing to engage in the treatment and remain longer in it (Hawley and Weisz, 2005; Flicker et al., 2008). Adding to that, Nobile and Drotar (2003) showed that effective parent-provider communication was associated with parental satisfaction, and adherence to treatment and communication was perceived as the most important factor of care for both parents and adolescents (Jager et al., 2015). Parental training may help to reach better parent-adolescent relationships (Ozturk et al., 2019). This theme contributed substantially to the insight on how important is to involve parents in the treatment and to create a good relationship with them.

### Parents as a barrier to effective care

Professionals recognized parents as the biggest barrier in the process of care, entailing, among other things, the diagnostic process, adherence to treatment and cooperation with other professionals. Previous studies brought similar findings (Kazdin et al., 1997a,b; Radovic et al., 2015). Parental fear of stigma was identified as one of the barriers, which is in line with previous research (Gulliver et al., 2010; Seamark and Gabriel, 2018). Moreover, a previous study (Richardson, 2001) found that parents had negative perceptions and expectations of mental health care providers and a lack of trust in them. These findings show the potential negative effects of negative perceptions and expectations on both sides. A further explanation is that parents are overwhelmed by the responsibilities of parenting and attending care (Owens et al., 2002). Considering all the findings mentioned above, we can conclude that not only adolescents (as clients of psychosocial care), but also their parents need the support of professionals.

### Overall perception of parents and their role in the care by care providers

Care providers perceive parents and their role in the care process mostly in a negative way (three of the four identified overarching themes) as either the source of adolescents´ problems, a barrier or a hindrance to providing care. However, besides perceiving parents negatively, professionals also understand parents and report positively about them as essential actors if they take an active role in the process of providing care to their children. Our findings raise important issues. The negative perceptions of professionals regarding parents can *per se* hinder effective cooperation with parents and can trigger the cycle of blame described by O'Sullivan and Russell (2006) with negative perceptions of parents towards care providers and negative perceptions of providers towards parents as not being involved and even being a barrier to the care. This can result in a mutual lack of trust that hinders the process of effective parent-provider cooperation. However, not all perceptions of care providers about parents were negative, with some reporting rather positively about the role of parents in care. This suggests the negative cycle of blame and overall mutual distrust can be broken and an effective parent-provider relationship can be established resulting in positive outcomes in form of better communication, more involved cooperation, and higher adherence (Nobile and Drotar, 2003; Hawley and Weisz, 2005; Flicker et al., 2008; Jager et al., 2015). This implies a need to identify and employ strategies to change the negative perspectives on both sides and establish good cooperation between parents and care providers, and subsequently, train care providers in their use.

### Strengths and limitation

A major strength of this study was its qualitative design, which provided the opportunity to bring detailed insight into the perceptions of psychosocial care providers on the role of parents in care. Moreover, by using the methodology of consensual qualitative research, we avoided the subjective personal perspective of the researcher, as all coders had to agree on codes for the analyzed data. The limitation of this study is that we may not have achieved the maximum saturation of professionals in terms of the number of interviews conducted. However, the sample selection was designed and able to reach a heterogeneous sample representative of the main types of psychosocial care providers. Also, during data collection, we reached a point of saturation, where no new themes occurred.

### Implications

Our findings have several implications for practice. First, professionals need education and training on how to work with multi-problem families, as many problems have their origin in that setting. Second, professionals also need support themselves, given the negative perception of some parents. These interventions should help reflect negative perceptions and attitudes towards parents and teach providers how to deal with them, with the aim of achieving unbiased care. Third, as providers understand the importance of the parental active role and the need to overcome barriers in parents, we need implementation and enhancement of patient-centered care principles. Our study also brought implications for future research. The care providers we interviewed did not refer to specific types of parents of adolescents in vulnerable groups who more likely to have emotional and behavioral problems, e.g., adopted youths or those in institutional care (Muzi and Pace, 2020) or parents of adolescents with specific psychiatric diagnoses (Di Trani et al., 2013). However, it might be of interest to address that in future. Also, more evidence is needed on which strategies professionals use to improve parent-care provider relationships and effectively involve parents in care and, and support patient-centered care. This may bring important findings and improvement in psychosocial care for adolescents. Last but not least, it might be of interest to look more in-depth into specific types of care and institutions and care providers involved in this care.

## Conclusion

Regarding the professionals’ perceptions of parental role in the care, we identified four main themes: 1. parents as a source of adolescents’ problems; 2. parents trying to escape from responsibility for adolescents with problems; 3. parents as an active part of the care for adolescents; and 4. parents as a barrier to effective care. There is a need for specific interventions aimed at supporting the involvement of parents in care, as their role is important. Moreover, the need for specific training on how to work with multi-problem families and the negative perceptions and attitudes of professionals towards parents has been raised.

## Data availability statement

The datasets presented in this article are not readily available to protect patient privacy. Requests to access the datasets should be directed to zuzana.dankulincova@upjs.sk.

## Ethics statement

The studies involving human participants were reviewed and approved by Ethics Committee of the Faculty of Medicine at P. J. Safarik University in Kosice. The patients/participants provided their written informed consent to participate in this study.

## Author contributions

ZV participated in design, coordination of the study, and data collection. JM conducted literature searches, provided summaries of previous research, and drafted the initial manuscript. JM, ZV, and AG worked on the analyses and interpretation of the data. ZV, AG, DJ, JvD, and SR provided the supervision, contributed with their comments to the manuscript, and approved its final version as submitted. All authors contributed to the article and approved the submitted version.

## Funding

This research was funded by the Slovak Research and Development Agency under the contract number APVV-15-0012 and APVV-21-0079, and Scientific Grant Agency of the Ministry of Education, Science, Research and Sport of the Slovak Republic and the Slovak Academy of Sciences, Reg. No. 1/0177/20.

## Conflict of interest

The authors declare that the research was conducted in the absence of any commercial or financial relationships that could be construed as a potential conflict of interest.

## Publisher’s note

All claims expressed in this article are solely those of the authors and do not necessarily represent those of their affiliated organizations, or those of the publisher, the editors and the reviewers. Any product that may be evaluated in this article, or claim that may be made by its manufacturer, is not guaranteed or endorsed by the publisher.
